# Highly Sensitive and Selective *In-Situ* SERS Detection of Pb^2+^, Hg^2+^, and Cd^2+^ Using Nanoporous Membrane Functionalized with CNTs

**DOI:** 10.1038/srep25307

**Published:** 2016-05-04

**Authors:** Mohamed Shaban, A. R. Galaly

**Affiliations:** 1 Nanophotonics and Applications (NPA) Lab, Department of Physics, Faculty of Science, Beni-Suef University, Beni-Suef, 62514, Egypt; 2 Department of Environment and Health Research, the custodian of the two Holy Mosques Institute for Hajj and Umrah Research, Umm Al-Qura University, Makkah, P.O. Box(6287), Saudi Arabia

## Abstract

Porous Anodic Alumina (PAA) membrane was functionalized with CoFe_2_O_4_ nanoparticles and used as a substrate for the growing of very long helical-structured Carbon Nanotubes (CNTs) with a diameter less than 20 nm. The structures and morphologies of the fabricated nanostructures were characterized by field emission- scanning electron microscopy (FE-SEM), energy dispersive X-ray (EDX), and Raman spectroscopy. By uploading the CNTs on PAA, the characteristic Raman peaks of CNTs and PAA showed 4 and 3 times enhancement, respectively, which leads to more sensitive Surface-Enhanced Raman Spectroscopy (SERS) substrates. For comparison, PAA and CNTs/PAA arrays were used as SERS substrates for the detection of Hg^2+^, Cd^2+^, and Pb^2+^. The proposed sensor demonstrated high sensitivity and selectivity between these heavy metal ions. CNTs/PAA sensor showed excellent selectivity toward Pb^2+^ over other metal ions, where the enhancement factor is decreased from ~17 for Pb^2+^ to ~12 for Hg^2+^ and to ~4 for Cd^2+^. Therefore, the proposed CNTs/PAA sensor can be used as a powerful tool for the determination of heavy metal ions in aqueous solutions.

Carbon Nanotubes (CNTs) are attracting much interest owing to their novel commercial optoelectronic applications such as flat panel displays, ultra-sensitive electromechanical and chemical sensors, and hydrogen storage devices^1^,^2^,^3^,^4^. Also, the applications of CNTs for the removal of hazardous pollutants from gas streams and aqueous solutions have been studied extensively because of their large specific surface area, highly hollow and porous structure, light mass density, and strong interaction with pollutant molecules^5^,^6^. A number of experimental studies have already been carried out about the adsorption of heavy metal ions on a single wall and multiwall CNTs^5^,^7^,^8^. CNTs have been synthesized by numerous techniques, such as arc discharge, laser ablation, plasma-enhanced, and chemical vapor deposition (CVD) of hydrocarbon gasses (methane, ethane, and acetylene) at rather high temperatures over a catalytic material^9^,^10^. The deposited CNTs may be metals, semiconductors, or dielectrics where the band gap of semiconducting nanotubes can be tuned by changing the tube diameter^11^.

Porous anodic alumina (PAA) is a hexagonal nanoporous membrane for the growth of highly ordered CNTs due to its easy fabrication, low cost, high thermal stability, and high controlability^12^. Deposition of CNTs inside the pores of PAA membranes has gained much attention in recent years due to their potential use as a surface-enhanced Raman scattering (SERS) substrate^13^. The position of CNTs on the membranes will show a significant influence on the properties of the structures, particularly for sensor applications. Multi-walled (MW) CNTs can be grown inside PAA membrane by CVD from transition metal nanoparticles (NPs) and nanowires (NWs) deposited inside its pores. Whereas, disordered CNTs can be grown by pyrolysis of the carbon feedstock gas on the PAA pore walls themselves^14^. The diameters of the CNTs are adjusted and matched the PAA pores. This approach is limited to PAA membranes with pores of diameter ≥50 nm due to the difficulties associated with the metal deposition in very narrow pores and with mass transport of gasses during CVD^15^. The strategy of depositing metal into narrow PAA pores and then widening the pores by etching the membrane was followed to grow CNTs with diameters of sub-50 nm^16^,^17^. By the electrodeposition of small amounts of Co into the PAA pores, CNTs with a diameter of 25 nm were grown from pores with diameters of 60 nm^15^. CNTs with diameters ranging from 7 to 17 nm can be grown by CVD when Fe(III) solution is deposited on the top of the electrodeposited Co depending on the Fe(III) concentration^16^. CNTs of diameters down to 5 nm can be grown on these templates when a uniform amount of Fe(III) is deposited inside each pore using the magnetic assist^18^. For an instant, the CNTs on the outer surface of the porous nanostructure have more possibility to react with the reactant than those inside. Thus, it is meaningful to load the CNTs upon the outer surface of the PAA membrane. Additionally, CNTs can be used to guide, enhance, emit, and modify optical fields, phenomena that can be used for novel and efficient applications such as sensors based on SERS^19^,^20^,^21^,^22^.

SERS is a surface-sensitive technique that enhances Raman scattering by molecules adsorbed onto certain specially prepared rough metal surfaces. The SERS-based sensor is one of the most important nano-optical-based detection strategies for chemical speciation of toxic heavy metal ions in water at trace levels. Tan *et al.*^23^ used 2-mercaptoisonicotinic acid (2 MNA)-modified gold nanoparticles (AuNPs) as SERS nanosensor to detect Hg^2+^ and Pb^2+^ ions with detection limits of 3.4 × 10^−8^ and 1.0 × 10^−7^M, respectively. Temiz *et al.*^24^ synthesized and used poly (propylene amine) dendrimers modified with 1,8-naphthalimide to detect heavy metal ions (Al^3+^, Sb^2+^, As^2+^, Cd^2+^, and Pb^2+^) by SERS for concentration ranges of 1 × 10^−6^ to 5 × 10^−4^ M. Wang *et al.*^25^ applied Ag@ Polyaniline (core–shell) nanocomposites as active SERS nanoprobes to some heavy metal ions (Pb^2+^, Cu^2+^, Hg^2+^, and Cd^2+^). This sensor showed high selectivity for Hg^2+^ ions with a detection limit of 1 × 10^−12^ M. Guerrini *et al.*^26^ reported the detection of Hg^2+^ and CH_3_Hg^+^ traces using a self-assembled monolayer of 4-mercaptopyridine (MPY) on highly active SERS and robust hybrid plasmonic materials formed by a dense layer of interacting gold nanoparticles anchored onto polystyrene microbeads. Li *et al.*^27^ summarized and provided an overview of recent achievements and challenges in the determination of heavy metal, organic pollutants, ions, and pathogens based on SERS. Shaban *et al.*^28^ introduced PAA membrane functionalized with hexagonal arrays of Au nanoparticles as SERS substrate for the detection of different heavy metals including Hg^2+^, Pb^2+^, and Cd^2+^ with nM concentrations. Li *et al.*^29^ demonstrated a stable and reliable SERS method for simultaneous Hg^2+^ and Ag^2+^ detection using triple Raman label-encoded gold nanoparticle trimers with LOD values of 1.69 and 1.71 pg mL^−1^, respectively. Frost *et al.*^30^ employed the metal-affinity properties of a citrate functionalized gold nanoparticle (AuNP) to detect Pb^2+^ ions based on SERS. This detector showed a linear behavior between 50 ng/L and 1000 ng/L. Wei *et al.*^31^ summarized the uses of gold and silver nanoparticles for the development of highly sensitive SERS-biosensors for the detection of organic or inorganic pollutants and pathogens. There are two mechanisms described in the literature for SERS enhancement: a chemical and electromagnetic enhancement. The electromagnetic (EM) effect is dominant and the chemical effect is contributing two order of magnitude improvement.

Despite the rapid development of SERS- based sensors, the high costs and reproducibility of sensor fabrication still impede their practical environmental applications. Development of low-cost, rapid, reproducible, and scalable detection platforms remains a strong demand. In this work, an efficient and simple method is proposed to coat the top surface of the PAA membrane with an array of CNTs of diameters ≤20 nm. The fabricated blank PAA and CNTs/PAA membranes are used as ultra-high sensitive sensors for the detection of traces concentrations of Hg^+2^, Pb^+2^, and Cd^+2^ ions in water. The sensing principle is based on *in-situ* SERS spectroscopy.

## Results and Discussion

### Morphologies and chemical compositions of PAA and CNTs- coated PAA membranes

**Figure 1:**
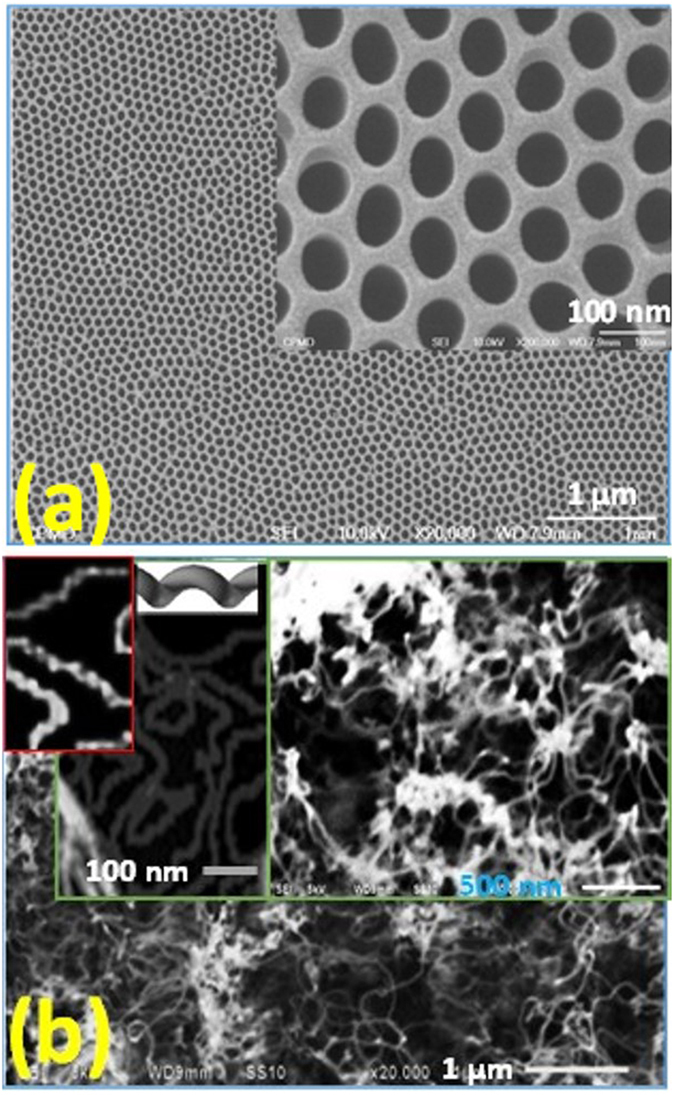
Top view FE_SEM image of (**a**) PAA membrane and (**b**) CNTs/PAA membrane anodized for 5 min and pore widened for 60 min.

The surface properties of the blank and CNTs- coated PAA membrane were characterized by FE-SEM. Figure 1(a) illustrates a typical top view FE-SEM image of PAA membrane anodized for 5 min and pore widened for 70 min. This figure shows nanopores aligned vertically with hexagonal distribution on the Al substrate. The pore diameter is approximately *D*_*p*_ = 70 nm, the interpore distance is *D*_*pp*_ = 100 nm, and the pore density is about 1.142 × 10^10^ cm^−2^. The porosity of the membrane is calculated by the relation 
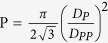
 to be 44.4%.

To improve the sensing properties of PAA membrane, CVD was used to grow CNTs on its top surface. Figure 1(b) shows top view FE-SEM images of CNTs/PAA membrane at different magnifications. As shown in this figure, the CoFe_2_O_4_ nanoparticles on the top surface of PAA have acted as seeds for growing very long CNTs of diameter 14–20 nm. The insets of Fig. 1(b) showed helical-structured carbon nanotubes with a large specific surface area. In addition to low cost and high stability of the fabricated helical CNTs/PAA, these novel structures represent a promising candidate as SERS substrates and may find a broad range of new applications, such as in sensors and environmental monitoring.

**Figure 2:**
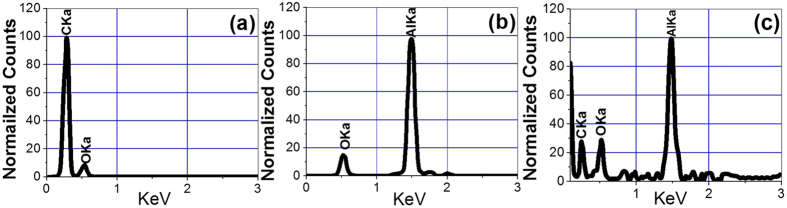
EDX spectra of (**a**) CNTs powder, (**b**) PAA membrane, and (**c**) CNTs/PAA.

The chemical composition of CNTs, PAA, and CNTs/PAA are investigated by energy dispersive X-ray (EDX) and shown in Fig. 2. The EDX spectrum of CNTs powder, Fig. 2(a), shows the signals of C and O elements. The quantitative results were 82.9% C and 17.1% O for CNTs. The EDX pattern in Fig. 2(b) illustrates the signals of Al and O elements. Quantitatively, the results were 52% Al and 48% O for Al_2_O_3_. The remained Al signal comes from the Al substrate. Figure 2(c) shows C, O, and Al signals for CNTs/PAA at percentages 16.9%, 17.7%, and 65.4, respectively.

### Raman Spectra of PAA, CNTs, and CNTs- coated PAA membranes

**Figure 3:**
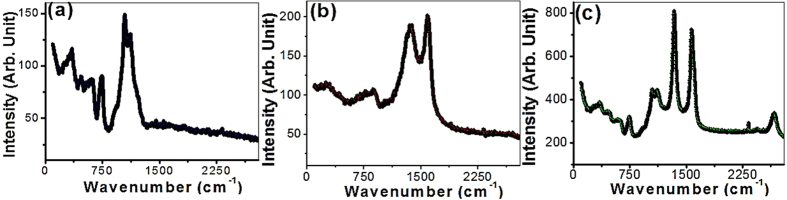
Raman spectra of (**a**) PAA, (**b**) CNTs powder, and (**c**) CNTs/PAA.

Figure 3 shows Raman spectra of (a) PAA, (b) MWCNTs, and (c) MWCNTs loaded on PAA membrane. As shown in Fig. 3(a), three characteristic peaks are illustrated at 750, 1050, 1118 cm^−1^. Two well-known first-order characteristic peaks for MWCNTs were observed at 1586 cm^−1^ (G band) and 1354 cm^−1^ (D band) as shown Fig. 3(b). The G band is related to the in-plane vibrations of sp^2^-bonded carbon atoms due to the graphitic nature of the nanotubes^32^. The D band is related to the presence of defects and disorder in the hexagonal lattice (carbonaceous impurities, broken sp^2^ bonds in the sidewalls, etc.)^33^. The D/G intensities ratio (I_D_/I_G_) indicates the level of defectiveness of the system’s graphitic lattice and measures the disordered atoms and defects. The calculated I_D_/I_G_ ratio is 0.955.

By uploading the CNTs on PAA, the characteristic Raman peaks of CNTs and PAA are shown in Fig. 3(c) with 4 and 3 times enhancement, respectively. Also, all the peaks are shifted to lower wavenumbers: 746, 1054, 1118 cm^−1^ for PAA peaks; and 1574, 1342 cm^−1^ for CNTs peaks. This shift indicates some reductions at PAA/CNTs interfaces during the growth of CNTs in N_2_ environment at the high temperature^34^. Furthermore, the increase in the I_D_/I_G_ value to 1.12 indicates that the disorders and defects of CNTs on PAA surface are greater than free standing CNTs. The high intensity of D band indicates the low crystallinity of the CNTs as confirmed by the high-resolution SEM images, Fig. 1(b). Upon the loading of CNTs on PAA, two additional emission peaks are observed at 2330 and 2774 cm^−1^. Moreover, the very broad background over the entire measurement range is due to luminescence from the CNTs/PAA membrane. The deposition of very long helical CNTs on the outer surface of PAA is important for sensing applications for three reasons. First, the CNTs on the outer surface of PAA has more possibility to touch the reactant. Second, the enhanced Raman characteristics and the higher surface area of CNTs/PAA membrane may enhance the sensing properties. Finally, the existence of the defects and disorders may act as additional hot spots. These reasons make this CNTs/PAA array fascinating SERS substrate. To explore that, PAA and CNTs/PAA arrays are used as selective and sensitive SERS substrates for the detection of heavy metals, especially Cd^2+^.

### Sensing properties and SERS measurements for PAA and CNTs/PAA membranes

SERS is a surface-sensitive technique that results in the enhancement of Raman scattering by molecules adsorbed on rough metal surfaces. SERS measurements are used to investigate the vibrational properties of the adsorbed molecules yielding structural information on the molecule and its local interactions. Thus, SERS measurements uniquely identify and enable the detection of individual^35^,^36^. The unique properties of CNTs/PAA nanostructure offer a new way to achieve high sensitivity and selectivity, as compared to the blank PAA and aqueous solution.

**Figure 4:**
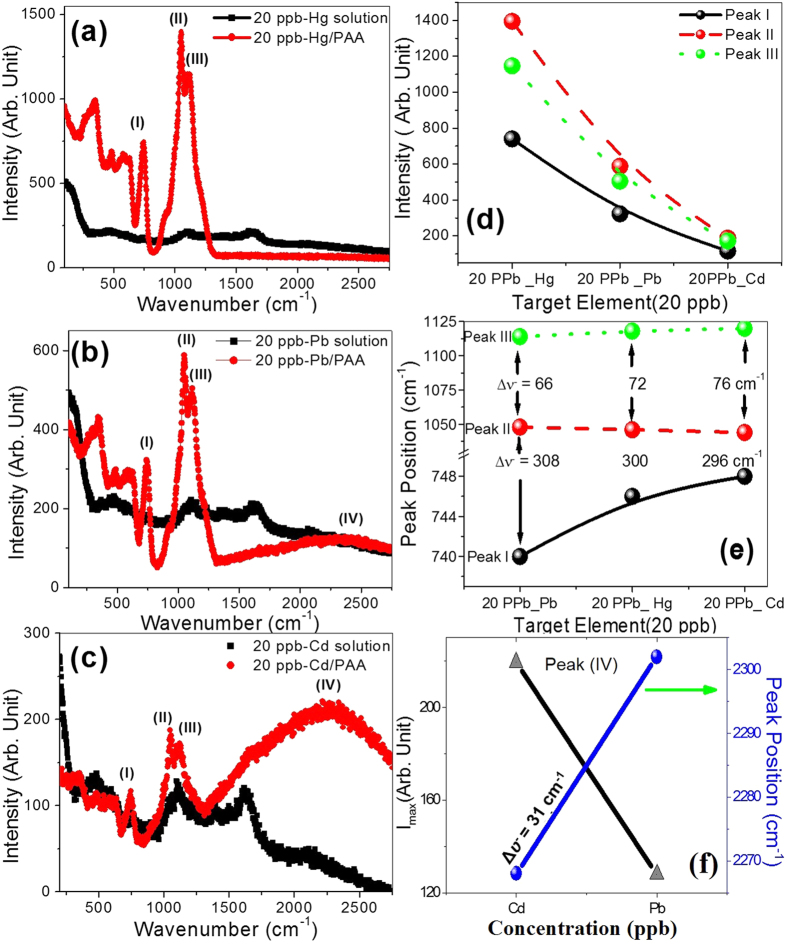
Demonstrating the selectivity of PAA membrane: Raman spectra of bare solutions and PAA membranes uploaded with (**a**) Hg^2+^, (**b**) Pb^2+^, and (**c**) Cd^2+^ of concentration 20 ppb; (**d–f**) the variation of intensity and position of the characteristic peaks, I, II, III, and IV for the different ions.

Selectivity and sensitivity of the blank PAA substrate are addressed to identify the role of PAA membrane as SERS substrate. Selectivity of PAA sensor is demonstrated in Fig. 4. Figure 4(a) shows SERS spectra of bare 20 ppb-Hg solutions and PAA membrane contaminated with 20 ppb -Hg^2+^ aqueous solution. Figure 4(a–c) shows SERS spectra of bare solutions and identical PAA membranes contaminated with 20 ppb- Hg^2+^, Pb^2+^, and Cd^2+^, respectively. As shown, the contaminated PAA membranes show intense and sharp SERS peaks compared to the collected data from the corresponding contaminated solutions. In contrast with the weak SERS of the bare Hg, Pb, and Cd solutions, the intensities of the characteristic peaks are almost 15, 3, and 1.5 fold for Hg^2+^, Pb^2+^, and Cd^2+^, respectively. Also, the contrast of the SERS peaks is much improved. For example, the contrast of peak II, the difference between the maximum and minimum intensity, is about 18, 8, and 2 times for Hg^2+^, Pb^2+^, and Cd^2+^, respectively, about that of the corresponding contaminated solutions after subtracting the background. Figure 4(d) shows the variation of the intensities of peaks I, II, and III for the different heavy metals and demonstrates a pronounced nonlinear decrease in the intensity. Also, upon the addition of heavy metals, peaks I and III are slightly shifted to higher wavenumbers and peak II is slightly shifted to lower wavenumbers as shown Fig. 4(e). The separation, Δῡ, between peaks II and III is increased from 66 to 76 cm^−1^ and between peaks I and II is decreased from 308 to 296 cm^−1^ for Pb^2+^/PAA and Cd^2+^/PAA respectively. In addition, a broad peak centered at 2268 cm^−1^ for Cd^2+^ and 2302 cm^−1^ for Pb^2+^ is observed. As shown in Fig. 4(f), the intensity of this peak is stronger with Cd^2+^ than Pb^2+^. Therefore, the result showed higher sensitivity toward Hg^2+^ than the other metal ions and reasonable selectivity toward Cd^2+^ and Pb^2+^. Relative to the corresponding bare solutions, the intensity can be increased from 10 to 100 orders of magnitude if PAA is used as SERS substrate. This may be ascribed to the chemical enhancement and more efficient hotspots, active nanodots with diameters less than 15 nm, at the surface of the PAA membrane^28^,^37^. The existence of anionic impurities in the structure of PAA makes the pore surface of PAA prone to attack by oxides to generate surface hydroxyl groups, which act as nucleation centers for its further covalent functionalization. Also, the interference of the reflected rays from the air/metal, metal/PAA, and PAA/Al interfaces may lead to the electromagnetic enhancement and significant signal amplification^37^. Then, the trapping of PAA with adsorbed heavy metals provides a performance improvement because of the increase in the number of analyte molecules in SERS-active hot spots and the electromagnetic enhancement due to the interference of optical waves within the interaction volume.

**Figure 5:**
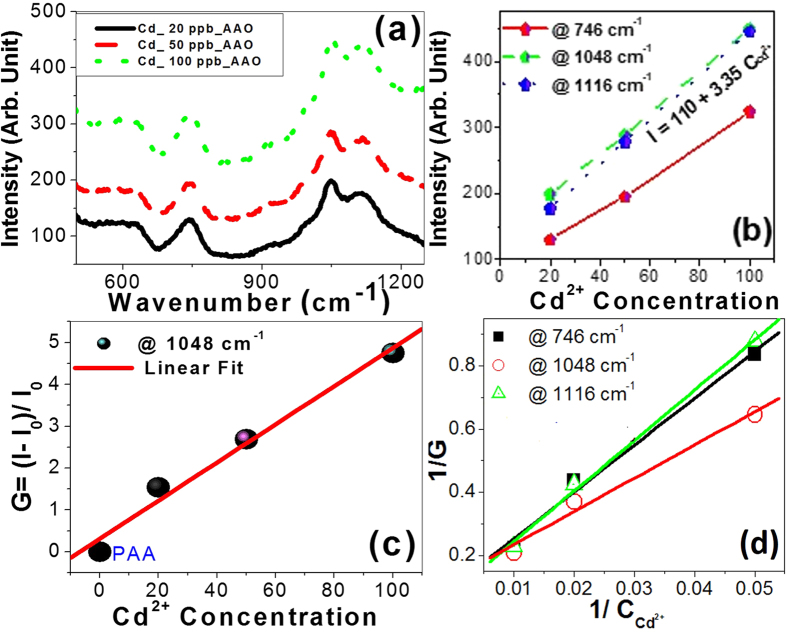
The sensitivity of PAA sensor: (**a**) Raman spectra, (**b**) peak intensity and (**c**) enhancement factor vs. Cd^2+^ concentrations, and (**d**) shows 1/G vs. 1/C_cd2+._

To check the sensitivity of the PAA sensor, the SERS spectra of the PAA were measured after the addition of various concentrations of Cd^2+^ ions (up to 100 nM), Fig. 5(a). As shown in Fig. 5(a,b), the intensity of SERS was very sensitive to the change in the Cd^2+^ concentration and increased by increasing the Cd^2+^ concentration. The derived normalized sensors response (normalized intensity change or enhancement factor, G) was quantified by the equation (1):


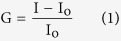


where I_o_ and I were the SERS intensity at particular wavenumber before and after Cd^2+^ addition on PAA membrane, respectively. A linear correlation of the G values with concentrations of Cd^2+^ ions was observed as shown in Fig. 5(c). The curve is linear and can be well fitted by equation (2):


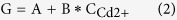


where A = 0.8954, B = 83.45, and C_Cd2+_ is the Cd^2+^ ions concentration. This relation has also been further confirmed by the linear fitting of (1/G) vs. (1/C_Cd2+_) as shown in Fig. 5(d). This behavior implies that the surface coverage of adsorbed molecules follows Langmuir isotherm. Therefore, there are no interactions between the adsorbed molecules on adjacent hotspots and each hotspot can hold at most one molecule.

**Figure 6:**
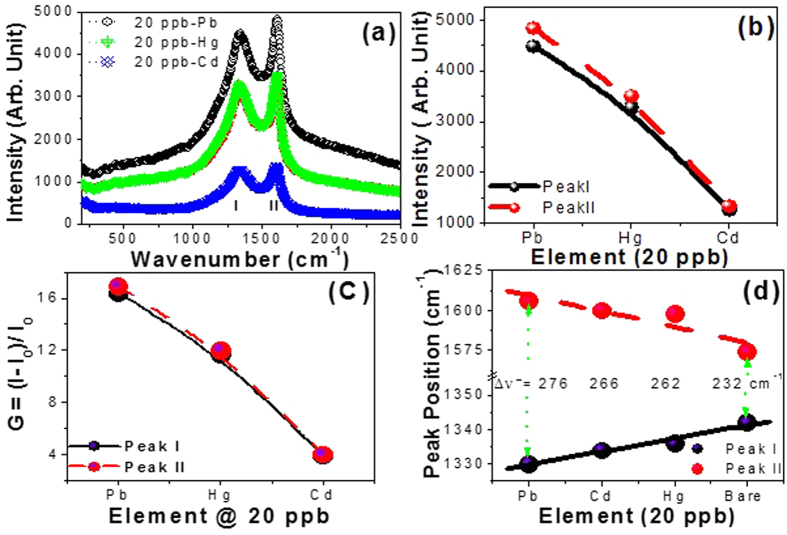
Demonstrating the selectivity of CNTs/PAA sensor: Raman spectra of CNTs/PAA sensor uploaded with Hg^2+^, Pb^2+^, and Cd^2+^ of concentration 20 ppb; (**b–d**) the variation of intensity, enhancement factor, and position of the characteristic peaks I and II for the different ions.

**Figure 7:**
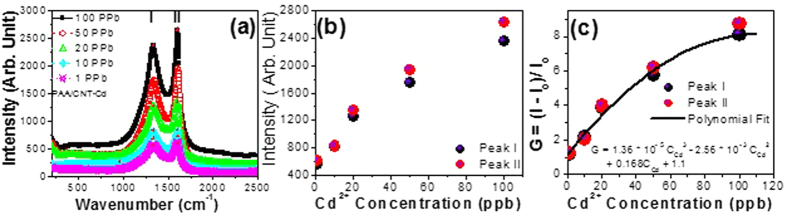
Demonstrating the sensitivity of PAA/CNTs sensor: (**a**) Raman spectra, (**b**) peak intensity and (**c**) enhancement factor vs. Cd^2+^ concentrations.

The sensing properties, selectivity and sensitivity, of CNTs/PAA sensor are demonstrated in Figs 6 and 7. Such selective response to Pb^2+^, Hg^2+^ and Cd^2+^ were collectively exhibited by the measured SERS spectra in Fig. 6(a). All the spectra in this Figure show only two intensive bands (I and II) related to CNTs. However, the other bands that are related to PAA are dismissed. This may be ascribed to the significant increase in the intensity of these two peaks upon the addition of heavy metal ions. The variation of the characteristic peaks intensity and enhancement factor are shown in Fig. 6(b,c). The SERS spectra show stronger peaks for Pb^2+^ than Hg^2+^ and Cd^2+^. The values of G relative to the peaks of bare CNTs are decreased from ~17 for Pb^2+^ to ~12 for Hg^2+^ and to ~4 for Cd^2+^. The values of G relative to the corresponding bare solutions are ~22, 16, and 10 for Pb^2+^, Hg^2+^, and Cd^2+^, respectively. Then, the uploading of CNTs upon PAA can increase the adsorption capacity and selectivity of the heavy metal ions. The unique adsorption property of CNTs/PAA offers a new way to achieve good selectivity, as compared to the direct measurements using CNTs or PAA only. The raw CNTs are rarely used for selective sorption of ion metals due to van der Waals interactions among carbon atoms in graphene sheets. In contrast, modified CNTs are better sorbents and more selective than raw CNTs for metal ions, because functionalization not only increases sorption metal ions through chemical bonding but also improves dispersion in the media^38^. Here, helical-structured carbon nanotubes with a large specific surface area are well distributed on the top surface of PAA as shown in Fig. 1(b) inset. Also, the stability constants of CNTs with Pb^2+^ are greater than with Hg^2+^ and Cd^2+ ^28,38. Therefore, CNTs/PAA will be able to show high selectivity and form much more stable complexes with Pb^2+^ than with other metal ions. Figure 6(d) displays the variation of the peak I and II positions for the different heavy metals. Upon the addition of heavy metals, peak I shifted to lower wavenumbers and peak II shifted to higher wavenumbers. These changes are more pronounced for Pb^2+^ than Hg^2+^ and Cd^2+^. Also, as shown in Fig. 6(d), the separation between peak I and II, Δῡ, is increased from 232 to 276 cm^−1^ for bare CNTs/PAA and Pb^2+^/CNTs/PAA, respectively. Hence, Δῡ/ΔC = 2.2, 1.7, and 1.5 cm^−1^/ppb for Pb^2+^, Hg^2+^, and Cd^2+^, respectively. Therefore, the results showed pronounced selectivity toward Pb^2+^ over other metal ions.

Figure 7 demonstrates the sensitivity of CNTs/PAA sensor for different concentrations of Cd^2+^ ranging from 1 to 100 ppb. This sensor exhibited a high sensitivity up to 1 ppb, which is the lowest detectable concentration in this study. The SERS spectra in Fig. 7(a) and the plot of SERS peak intensity versus the Cd^2+^ concentration (Fig. 7(b)) clearly show a significant nonlinear increase in the detected characteristic peaks with increasing the Cd^2+^ concentration. Also, the G values are calculated and illustrated in Fig. 7(c). The curve is nonlinear and can be well fitted with the following third order polynomial equation (3):


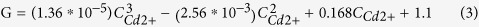


In contrast to typically CNTs, helical CNTs are composed of graphene sheets that stack upon each other, to produce unique 3D helical nonlinear morphology from hollow and solid carbon structures due to the existence of the consecutive knees of a graphene plane. These helical CNTs possessed electrical conductivities of 30–50 S/cm depending on the surrounding temeperature^39^. Also, 3D electron hopping model was proposed to explain the mechanism of electron transport within these CNTs. Using this model the electron hopping length can be adapted from ∼5 to 50 nm. Because our nanostructures sizes are ≤this hoping length, then these surfaces can effectively adsorb more metal ions than the typical CNT or PAA surface. According to our results, the following order of adsorption of the metal ions on the active sites of the 3D helical CNTs is observed: Pb^2+>^ Hg^2+>^ Cd^2+^. Similar results are shown for functionalized single-walled CNTs with -COO(-), -OH, and -CONH_2_ groups^40^. Therefore, the trapping of CNTs/PAA sensor with the adsorbed heavy metals provides a performance improvement. This improvement is ascribed to the increase in the number of analyte molecules in SERS-active hot dots, the existence of the trapping electrons, the nanoporous nature of the surface morphology, in addition to the excitation and interference of optical waves within the interaction volume. Then, uploading CNTs on PAA enables the proposed sensor to have a wide linear range of concentrations, high sensitivity, good selectivity and reproducibility for the determination of these heavy metal species.

## Conclusion

Here, we introduced a new type of SERS sensor with high sensitivity and selectivity for the determination of heavy metals (Pb^2+^, Hg^2+^, and Cd^2+^) at ppb concentrations. Very long helical- CNTs with diameter <20 nm were grown by CVD on the surface of PAA membrane functionalized with CoFe_2_O_4_ nanoparticles. The sensing property of PAA and helical- CNTs/PAA nanosensors was systematically studied. CNTs/PAA has been demonstrated as a more reliable and sensitive heavy metals sensor compared to the bare PAA membrane. The CNTs/PAA sensor showed a high sensitivity up to 1 ppb, which is the lowest detectable concentration at room temperature in this study. The values of normalized sensor response, G, relative to the corresponding bare solutions are ~22, 16, and 10 for Pb^2+^, Hg^2+^, and Cd^2+^, respectively. The selective sensing to Pb^2+^, Hg^2+^, and Cd^2+^ can be achieved, respectively, in the CNTs/PAA sensors. The proposed CNTs/PAA sensor possess direct and high sensitivity, high stability, good selectivity, low fabrication cost, cheap monitoring, and simplicity. Then the current sensor is a powerful tool for the on-site determination of heavy metal ions by a portable Raman spectrometer.

## Methods

### Functionalization of PAA membrane with CoFe_2_O_4_ nanoparticles

PAA membrane was prepared by a combined method from two-step anodization and pore widening method^41^. The first and second anodization processes were performed in 0.3 M oxalic acid at 40V and 9 °C for 180 and 5 min, respectively. The barrier layer was thinned by a successive drop in the DC voltage from 40 to 15 V at a rate of 0.1 V/s, and then maintained at 15 V for 5 min^41^. The pore widening was carried out for 70 min^37^. The widened PAA membrane was functionalized and covered with CoFe_2_O_4_ nanoparticles. 0.5M Fe (NO_3_) and 0.5 M Co (NO_3_)_2_.6H_2_O were mixed at 1:1. Then, the PAA membrane was immersed in the solution and left for 1 h in the ultrasonic. The obtained membrane was dried in an oven at 500 °C for 1 h.

### Growth of CNTs on functionalized PAA membrane

The C_2_H_2_ chemical vapor deposition (CVD) was carried out in a ceramic tube equipped with a gas flow and temperature controller. The nitrogen (N_2_) gas was passed for 10 minutes over the CoFe_2_O_4_ /PAA membrane at 600 °C. N_2_/C_2_H_2_ (5:1 V/V) mixture was then introduced into the system for 50 min. The N_2_ gas continuously flowed through the reaction chamber until the temperature of the tube furnace dropped. The prepared CNTs/PAA membrane was dried in an oven at 120 °C.

### Characterization Techniques

Morphological studies of the fabricated nanostructured films were carried out using field emission- scanning electron microscopy, FE-SEM (model: ZEISS SUPRA 55 VP and ZEISS LEO, Gemini Column). The chemical compositional analysis was explored using energy dispersive X-ray (EDX; Oxford Link ISIS 300 EDX). To obtain accurate quantitative analysis, standard additions were used to calibrate the response.

### SERS measurements

After injecting tiny amounts (=0.1 μl) of water contaminated with heavy metals on the surface via a microsyringe, the sensing principle is based on heavy metals detection by using surface-enhanced Raman scattering (SERS) spectroscopy. The SERS measurements were performed over the PAA and CNTs/PAA samples by an Enwave Optronics Raman microscope with 514 nm- excitation wavelength and a 1 μm spot size. The excitation laser power and the exposure time were P = 50 mW and t = 20 s, respectively. For comparison, the measurements were also carried out for Pb^2+^, Hg^2+^, and Cd^2+^ bare solutions.

## Additional Information

**How to cite this article**: Shaban, M. and Galaly, A. R. Highly Sensitive and Selective *In-Situ* SERS Detection of Pb^2+^, Hg^2+^, and Cd^2+^ Using Nanoporous Membrane Functionalized with CNTs. *Sci. Rep.* **6**, 25307; doi: 10.1038/srep25307 (2016).
